# A new seven level boost-type ANPC inverter topology for photovoltaic applications

**DOI:** 10.1038/s41598-021-01669-6

**Published:** 2021-11-18

**Authors:** Jagabar Sathik M., Dhafer J. Almakhles

**Affiliations:** Renewable Energy Lab, College of Engineering, Prince Sultan University, Riyadh, Saudi Arabia

**Keywords:** Electrical and electronic engineering, Photovoltaics

## Abstract

Developing of new photovoltaic inverter topologies is received more attention in the last few years. In particular, designing an active neutral-point-clamping inverter type structure is quite popular for PV applications. The output voltage is always half of the input voltage (*v*_*in*_), which further increases the voltage rating of dc-link capacitors in the conventional three-level ANPC. To rectify the above problem and increase the output voltage by reducing dc-link capacitors voltage rating, a new boost type seven-level ANPC inverter topology is proposed. The proposed topology consists of seven switches and one floating capacitor. The floating capacitor voltage is self-balanced, and the output voltage is 1.5 times higher than the input voltage. A detailed comparison for some power components, power loss and cost with other existing topologies are presented. Further, the proposed topology is validated in a prototype hardware setup for different load values.

## Introduction

The photovoltaic (PV) inverter structure is considerably simple yet highly efficient because the researchers develop a new design with fewer components and compact size. Among the various existing PV inverters, the transformerless (TL) inverter has more advantages like single-stage operation, no bulky transformers and less leakage current. The PV-TL inverters start from a few hundred to kilowatts ranges. Nevertheless, the novel topologies are often developed for single-phase grid-connected systems, more suitable for rooftop utility PV applications. It is worth mentioning that the TL inverters with the Switched-Capacitor Multilevel Inverter (SCMLI) topologies are paid more attention among the researchers to generating a high number of voltage levels with reduced switches and *dv/dt* stress^1–3^. The researchers develop a new SCMLI topology to produce better efficiency compared to other existing SCMLI topologies. However, in ANPC type topologies, output voltage (*vo*) is always half of the input voltage (*v*in) due to the mid-point clamping of the dc-link capacitors and the load, which increases the voltage rating of dc-link capacitors. Thus, to reduce the voltage rating and size of the dc-link capacitors, the floating capacitors (FCs) are used as a voltage multiplier to boost the output voltage. Many switched capacitor topologies are presented in the literature, and few are discussed here. In Ref.^4^, a new switched capacitor topology with a high inductive load is proposed for a fundamental frequency of 1 kHz. This topology produces a 7L level output voltage with a gain of 1:3. It also can be extended to the "*m*" level. Another topology with the same structure in which few IGBTs are replaced with power diodes is presented^5^. In Ref.^6^, a hybrid SCMLI is presented with reduced switches and the possibility of generating *m* number of levels. A newly developed topology in Ref.^7^ reduces switch count and can be extended by cascading the proposed basic unit.

**Figure 1:**
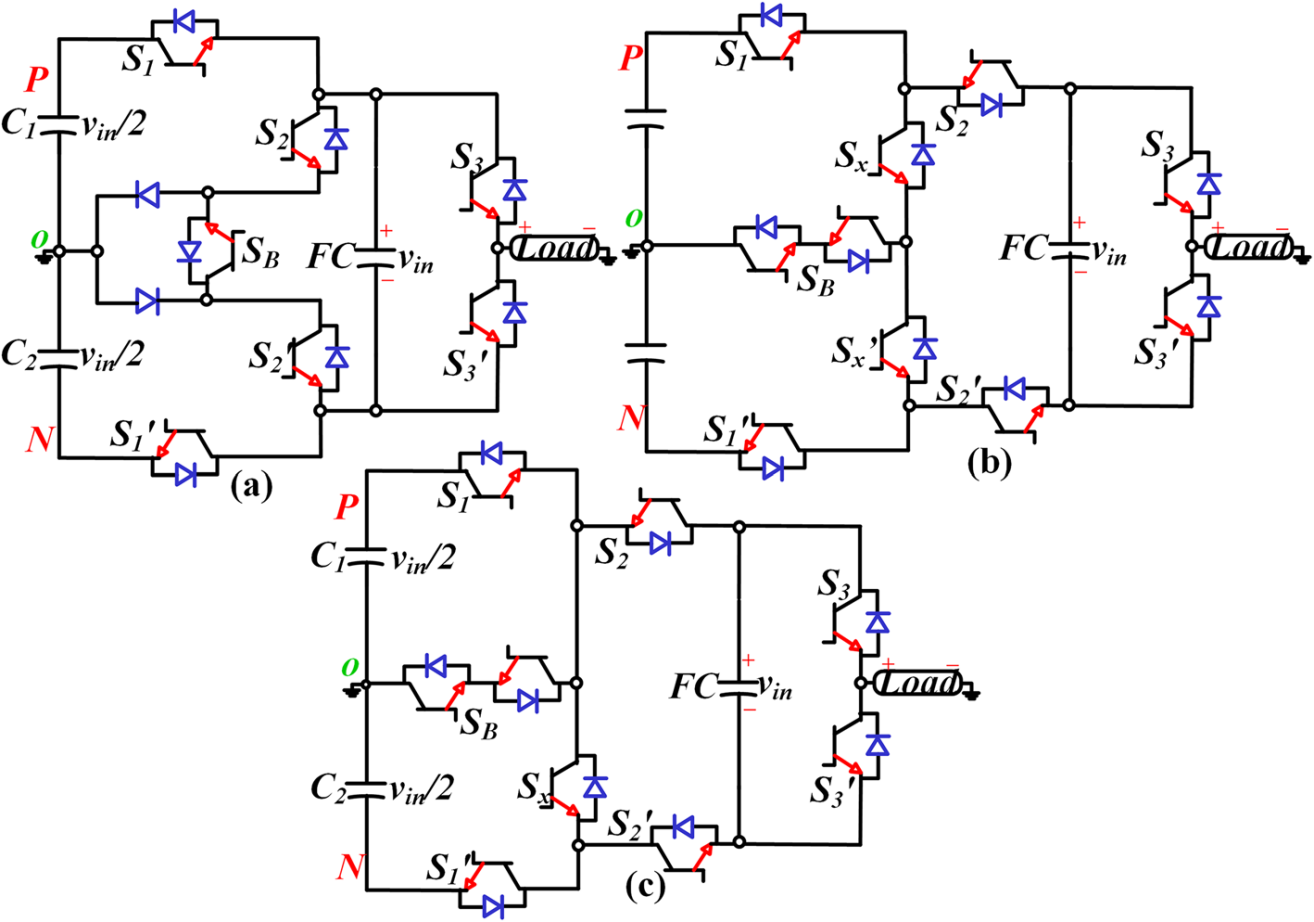
Boost ANPC type inverter topologies (**a**) 5L-ANPC topology presented in Ref.^7^, 7L-ANPC topology presented in (**b**)^9^, and (**c**)^13,14^.

The topologies presented in Refs.^4–8^ have the advantage of self-voltage balancing and boosting capability with a maximum of gain 1:3. However, these topologies needed a separate isolated dc source for a three-phase inverter system due to the non-availability of a common dc bus. Further, the stress on the switches is equal to 3*v*_*in*_. A single floating capacitor with ten IGBTs is used in Ref.^9^. The output voltage gain is 1.5 times higher than the input voltage. A new seven-level inverter topology with a logic form equation is proposed in the Ref.^10^. The rating of the floating capacitor voltage is *v*_*in*_/4. This topology needs an additional sensor to balance the capacitor voltage, increasing the inverter's complexity. New self-balanced neutral point clamped type SCMLI topologies are presented in Refs.^11–14^. A new 5L ANPC type inverter topology with a voltage boosting gain of 1:1 is presented in Ref.^10^ to overcome these challenges (see Fig. 1a). In this, seven switches and one floating capacitor is used. A new boost-type switched ANPC inverter topology with two floating capacitors is proposed in Ref.^11^. In this topology, the number of switches is ten, and this needs ten driver circuits as per the presented modes of operation. Furthermore, when the modulation index is less than or equal to 0.66, the upper floating capacitor always charges and has no path for discharge. A new high gain 7L inverter topology with ten switches and one floating capacitor is proposed in Ref.^12^ to avoid this overcharging of the upper capacitor under low modulation index, as shown in Fig. 1b. The and the improved structure of Ref.^12^ is presented in Refs.^13,14^ with a reduction of one switch, but still, the switch count is high, as shown in Fig. 1c. Six-switch seven-level inverter topology with a gain of three is recently reported in Refs.^15,16^. This topology uses a fewer number of switches and diodes. However, the number of capacitors is increasing and also the voltage stress on the capacitor is high. Further, the capacitors having low reliability as compared to other power components.

From the above literature, the SCMLI topology with voltage boosting ability is presented with many switches that increase the inverter's cost and size. However, the ANPC type topologies^10–14^ have the maximum blocking voltage of *v*_*in*_, which is one of the significant advantages of these topologies. In this letter, a new 7L ANPC topology is presented. The following points summarize the advantages of the proposed topology:

1. the conventional NPC and ANPC topologies output voltage is half of the input voltage, which is rectified, and the output voltage is boosted to be 1.5 times higher than the *v*_*in*_,
2. the FC has self-voltage balancing,
3. due to a smaller number of components, it features reduced power losses and cost of the inverter,
4. the maximum voltage stress on the switch is *v*_*in*_, i.e. 2/3rd of output voltage and
5. the number of switches in high current stress is two.

This article aims to prove the operation of the proposed topology with supporting evidence of the experimental validation. The proposed topology performance is observed during the various dynamic changes of external parameters like dc input voltage variations, load variations and internal variation of modulation index (*Ma*).

## Proposed self-balanced and boost (RSC-SB2) type 7L-ANPC inverter topology

### Circuit descriptions

**Figure 2:**
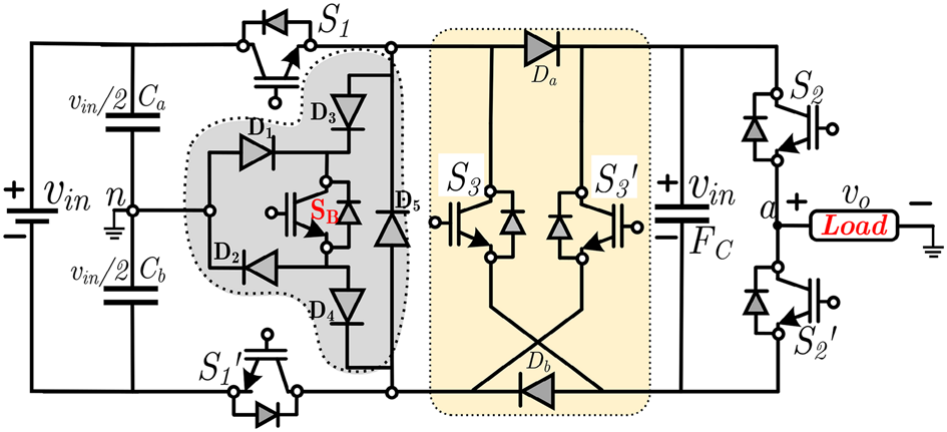
Circuit diagram of proposed 7L ANPC inverter topology.

Figure 2 shows the circuit diagram of the proposed ANPC type 7L inverter. The proposed circuit diagram comprises two dc-link capacitors (C_1_ and C_2_), six switches (S_1_, S_1_′, S_2_, S_2_′, S_3_, and S_3_′), one bidirectional switch (S_B_), six diodes and one floating capacitor (FC). The switch S_1_ and S_1_′ are connected with upper and lower dc-link capacitors. Further, the mid-point of the dc-link capacitors is connected to the negative terminal of the load and bidirectional switch (S_B_). Therefore, the dc-link capacitors are directly connected to the dc input voltage (v_in_), and these capacitors share the input voltage to v_in_/2. Furthermore, since the mid-point of the dc-link capacitor is connected directly to the load, it maintains the voltage (v_in_/2) itself.

### Modes of operations

**Figure 3:**
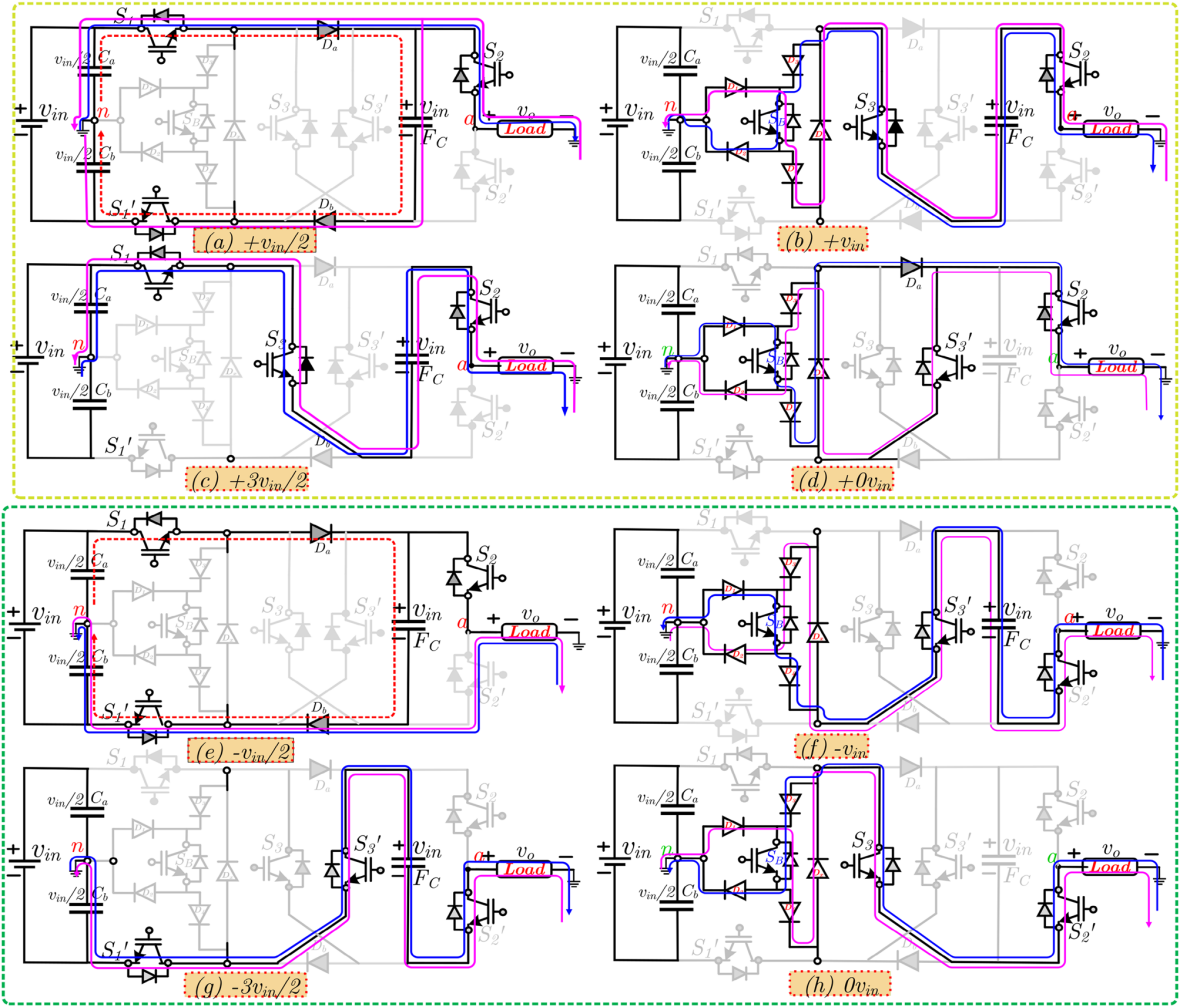
Modes of operation of proposed 7L ANPC inverter topology (**a**–**d**) positive half cycle and (**e**–**h**) negative half cycle.

Figure 3a–h for both positive and negative half cycle, the current path for various output voltage generation levels. From Fig. 3, the ON state switches are highlighted in a dark-black line.

The upper dc-link capacitor is connected with load for the positive half cycle, and in the negative half cycle, the bottom dc-link capacitor is connected to the load. A detailed explanation of each mode of operation is as follows:

+ *v*_*in*_/2: *S*_1_*, D*_*a*_*, D*_*b*_ & *S*_1_′ the switches are turned ON to charging the FC to *v*_*in*_ and simultaneously, the *S*_2_ is turned ON to supply the + *v*_*in*_/2 to the load.
+ *v*_*in*_: *S*_*B*_,* D*_*a*_,* S*_3_ and *S*_2_ the switches are turned ON to discharging the FC voltage to load. Now, the load voltage is equal to FC stored value (+ *v*_*in*_)*.*
+ 3*v*_*in*_/2: *S*_1_, *S*_3_ and *S*_2_ switches are turned ON, and the upper dc-link capacitor is the positive terminal connected to the negative terminal of FC. Now, the load voltage is equal to the sum of the C_1_ and FC i.e. *v*_*o*_ = + 3*v*_*in*_/2*.*
± 0 *v*_*in*_: in zero levels, the topology provides two redundant paths as either S_B_, S_3_ & S_2_′ or S_B_, S_3_′ and S_2_ switches turned ON.
− *v*_*in*_/2: *S*_1_,* D*_*a*_,* D*_*b*_ and *S*_1_′ the switches are turned ON to charging the FC to *v*_*in*_ and simultaneously, the S_2_′ is turned ON to supply the − *v*_*in*_/2 to the load.
− *v*_*in*_: *S*_*B*_,* D*,* S*_3_′ and *S*_2_′ the switches are turned ON to discharging the FC voltage to load. Now, the load voltage is equal to FC stored value (− *v*_*in*_)*.*
− 3*v*_*in*_/2: *S*_1_′_,_ *S*_3_′ and *S*_2_′ switches are turned ON, and the bottom dc-link capacitor is the negative terminal connected to the positive terminal of FC.

Now, the load voltage is equal to sum of the *C*_2_ and FC i.e. *v*_*o*_ = − 3*v*_*in*_/2*.* The Diode *Dx* provides the current path during the lagging or leading power factor*.* The above discussion clearly shows that the proposed topology uses fewer ON state switches in each voltage level. The stress analysis on the switches is the important parameter for capacitor self-balanced inverter topologies. The high inrush current will occur during the parallel connection of FC and *v*_*in*_. To prevent the inrush, current the small inductor can be added to the circuit loop. The switched capacitor circuits facing a high inrush current, which is a notable drawback. To reduce the inrush current, a current limiting inductor is used. The inductor size is small, limiting the high inrush current to the required current^15^. The mathematical expression for the current limiting inductor is given in Eq. (1).

1 $$i_{ind} = \frac{1}{2}\sqrt {\frac{{C_{f} }}{{L_{ind} }}} \Delta V_{Cf}$$

where the *i*_*ind*_ is the maximum inrush current or loop current during the charging the FCs, L_ind_ is inductor value, and *C*_*f*_ is FC capacitance value. The charging current i.e. FC current four to five times higher than the load current. The voltage and current stress for the individual switch are given in Tables 1 and 2, respectively. It confirms that the proposed topology's maximum voltage stress is equal to *v*_*in*_ and current stress is *i*_*o*_ + *i*_*c*_ occurred in only two switches. Other topologies presented in Refs.^8–11^ needed four switches with high current stress.

**Table 1:** Voltage stress on switches.

Level	S_1_	S_1_′	S_2_	S_2_′	S_3_	S_3_′	S_B_	D_a_	D_b_
L_1_^+^	–	–	–	*v*_*in*_	*v*_*in*_	*v*_*in*_	*v*_*in*_/2	–	–
L_2_^+^	*v*_*in*_	*v*_*in*_	–	*v*_*in*_	–	*v*_*in*_	–	*v*_*in*_	*v*_*in*_
L_3_^+^	–	*v*_*in*_	–	*v*_*in*_	–	*v*_*in*_	*v*_*in*_/2	*v*_*in*_	*v*_*in*_
L_0_^±^	*v*_*in*_	*v*_*in*_	*v*_*in*_	–	–	*v*_*in*_	–	*v*_*in*_	*v*_*in*_
L_1_^−^	–	–	*v*_*in*_	–	*v*_*in*_	–	*v*_*in*_/2	–	–
L_2_^−^	*v*_*in*_	*v*_*in*_	*v*_*in*_	–	*v*_*in*_	–	–	*v*_*in*_	*v*_*in*_
L_3_^−^	*v*_*in*_	–	*v*_*in*_	–	*v*_*in*_	–	*v*_*in*_/2	*v*_*in*_	*v*_*in*_

**Table 2:** Current stress on switches.

Level	S_1_	S_1_′	S_2_	S_2_′	S_3_	S_3_′	S_B_	D_a_	D_b_
L_1_^+^	*i*_*o*_ + *i*_*c*_	*i*_*o*_ + *i*_*c*_	+ *i*_*o*_	–	–	–	–	*i*_*o*_ + *i*_*c*_	*i*_*o*_ + *i*_*c*_
L_2_^+^	–	–	+ *i*_*o*_	–	+ *i*_*o*_	–	+ *i*_*o*_	–	–
L_3_^+^	+ *i*_*o*_	–	+ *i*_*o*_	–	+ *i*_*o*_	–	–	–	–
L_0_^±^	–	–	–	± *i*_*o*_	± *i*_*o*_	–	± *i*_*o*_	–	–
L_1_^−^	*i*_*o*_ + *i*_*c*_	*i*_*o*_ + *i*_*c*_	–	− *i*_*o*_	–	− *i*_*o*_	*-*	*i*_*o*_ + *i*_*c*_	*i*_*o*_ + *i*_*c*_
L_2_^−^	–	–	–	− *i*_*o*_	–	− *i*_*o*_	− *i*_*o*_	–	–
L_3_^−^	–	− *i*_*o*_	–	− *i*_*o*_	–	− *i*_*o*_	–	–	–

## Modulation technique and comparison of recent 7L SCMLIs

**Figure 4:**
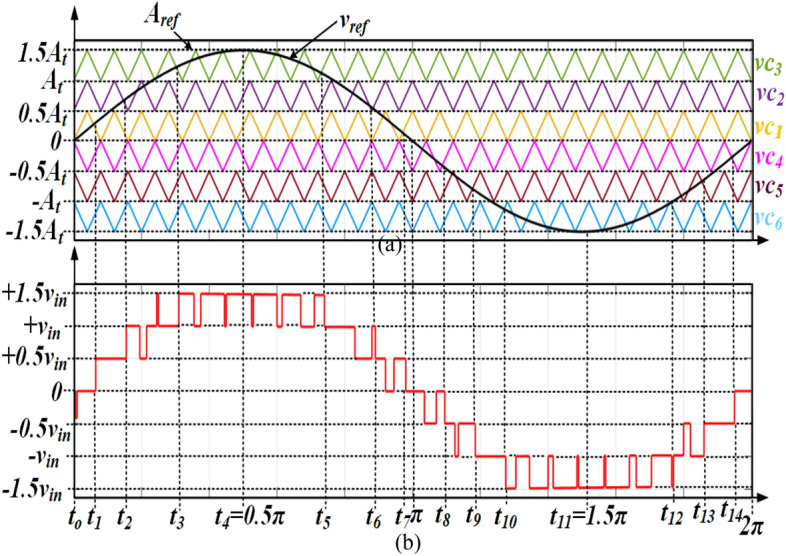
Modulation Technique (**a**) PD-PWM and (**b**) corresponding 7L waveform.

A variety of modulation methods such as selective harmonic elimination (SHE) PWM, multi-carrier PWM and can be applied to MLI. The SHEPWM can remove specific lower order harmonics. Similar to the SHEPWM, the pulse width modulation with phase disposition (PD-PWM) gives the overall lower THD compare to SHEPWM. In PD-PWM, the reference signal is compared with the carriers to generate the gate signals for each switch over a fundamental period. As shown in Fig. 4a, three triangular carriers *vc*_1_–*vc*_6_ with the same frequency, phase and amplitude are arranged from top to bottom in series, compared to sinusoidal *v*_*ref*_. Figure 4b demonstrates the 7L output voltage of the PD-PWM modulation for the proposed topology. According to Fig. 4, the PWM pulses for all switches are generated from the carrier and reference signal comparison. Longest Discharging Cycle (LDC) during the positive half-cycle occurs for FC_1_ during the time interval [t_2_–t_6_] and in the negative half-cycle for FC_1_ during the time interval [t_10_–t_12_]. The ripple value (*ΔV*_*rip*_) across each capacitor is shown in Eq. (2) as *R*_*o*_ is the resistive load and *f*_*o*_ (fundamental frequency) is the output voltage frequency. The optimum value for each capacitor (*C*_*opt*_) can be given as in Eq. (3).

2 $$\Delta V_{rip} = \frac{{1.5v_{in} }}{{2\pi f_{o} R_{o} C}}(t_{6} - t_{2} )$$

3 $$C_{opt} = \frac{{1.5v_{in} }}{{2\pi f_{o} R_{o} \Delta V_{rip} }}(t_{6} - t_{2} )$$

The proposed topology with other recent SCMLI topologies of both NPC and non-NPC types are compared in Table 3. From the comparison, the non-NPC topologies need high voltage and current stress switches. In a family of NPC types, topologies use more switches with high current stress except for the proposed topology. As compared to Refs.^4–14^, the proposed topology has low TSV and increased efficiency as compared to other topologies.

**Table 3:** Components comparison of recent 7L boost ANPC type SCMLI topologies.

	Refs.	A	B	C	D	E	F	G	H	I (µF)	J	K	L
Non-NPC type	^4^	10	–	2	3.0	4	3 *v*_*in*_	20.0	–	147	2.00	85.9/1 kHz	No
	^5^	8	2	2	3.0	2	3 *v*_*in*_	16.0	3.0	2200	2.00	NA/50 Hz	
	^6^^a^	7	4	2	3.0	1	3 *v*_*in*_	16.0	4.0	4700	2.29	92.9/50 Hz	
	^7^	8	2	2	3.0	2	3 *v*_*in*_	16.0	2.0	470	2.00	97.1/400 Hz	
	^8^	10	–	1	1.5	5	*v*_*in*_	9.0	–	4400	0.90	NA/50 Hz	
	^9^^b^	9	–	1	1.5	–	*v*_*in*_	7.0	–	2700	0.78	NA/50 Hz	
NPC type	^11^	10	–	2	1.5	4	*v*_*in*_	9.0	–	4700	0.90	97.0/50 Hz	Yes
	^12^	10	–	1	1.5	4		9.5	–		0.95	97.0/50 Hz	
	^13^	9	–	1	1.5	4		8.5	–		0.94	96.7/50 Hz	
	^14^	9	–	1	1.5	4		8.5	–	1000	0.94	95.8/50 Hz	
	Pro	7	6	1	1.5	2	*v*_*in*_	6.5	5.0	2200	0.93	97.3/50 Hz	

A—number of switches, B—number of diode, C—number of floating capacitor, D—output voltage gain, E—number of switches in *i*_*o*_ + *i*_*c*_, F—maximum voltage stress of FC, G—total standing voltage (TSV) × *v*_*in*_ for switches, H—total standing voltage (TSV) × *v*_*in*_ for diode, I—FC capacitance value, J—TSV/N_Switches_, K—efficiency (%)/fundamental frequency and L—3Φ-inverter with single dc source/link, NA—not addressed.

^a^The huge inrush current will occur on a single switch due to the charging of all the capacitors at the same time.

^b^Additional sensors are required to balance the floating capacitors.

## Results and discussions

**Figure 5:**
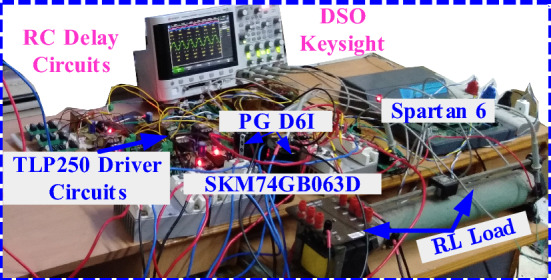
Prototype hardware setup.

**Figure 6:**
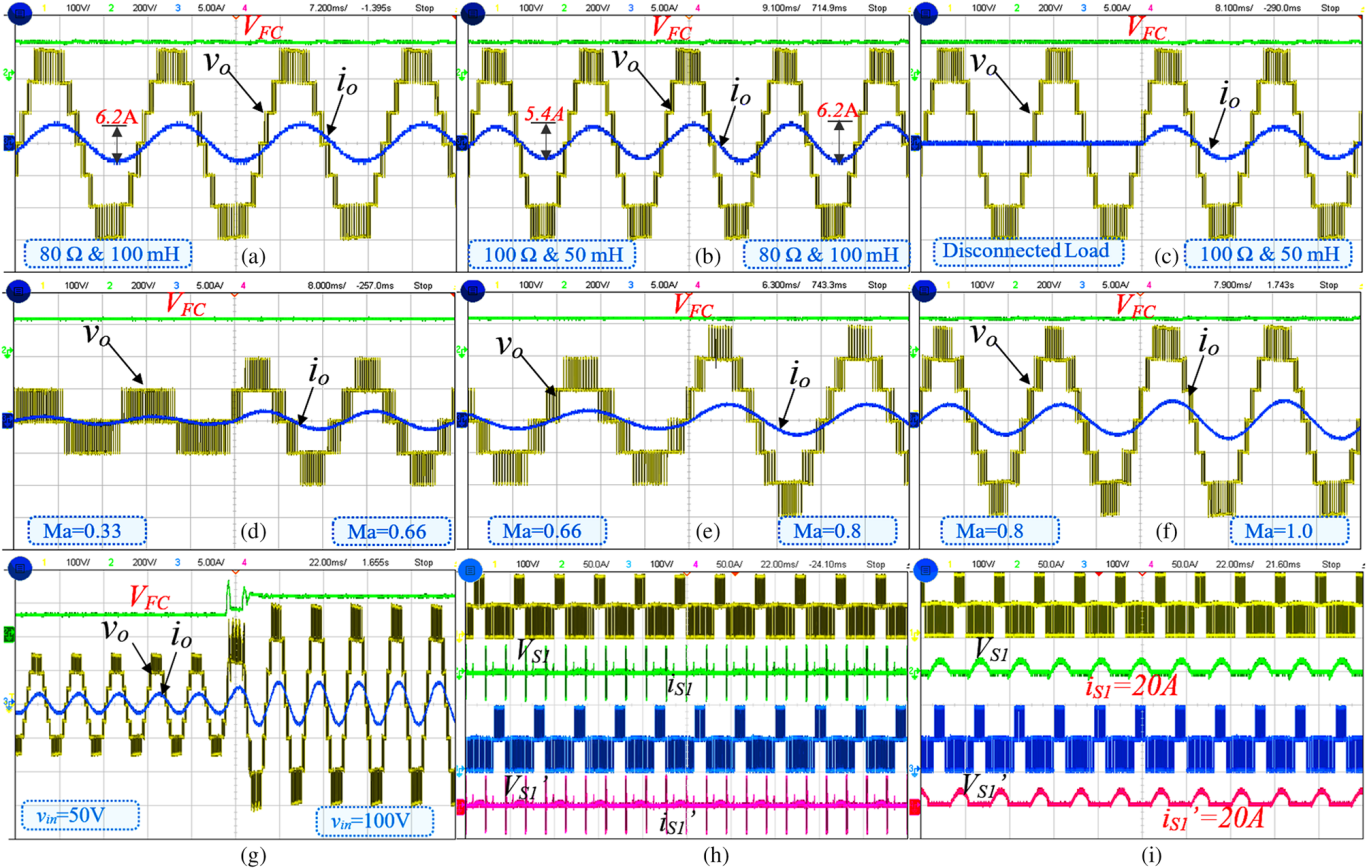
Experimental results of proposed 7L boost type ANPC Inverter, (**a**) output voltage and current, (**b**) sudden load changes from 100 Ω and 50 mH to 80 Ω and 100 mH, (**c**) no-load to 100 Ω and 50 mH, modulation index (Ma) variations (**d**) 0.33–066, (**e**) 0.66–0.8, (**f**) 0.8–1.0, (**g**) sudden input voltage changes from 50 to 100 V, Switch (S_1_ and S_1_′) voltage and current (**h**) without loop inductor and (**i**) with loop inductor.

The scaled-down experimental setup is fabricated for the real-time implementation of the proposed topology, as shown in Fig. 5. The detailed components list and corresponding ratings are summarized in Table 4. Finally, the practical validation for the various condition is tested and the corresponding values are measured as shown in Fig. 6a–i. Initially, the typical resistive and inductive load with values of 80 Ω and 100 mH is applied, and the results are shown in Fig. 6a with the maximum output voltage of 300 V and the current value of 3.1 A for *v*_*in*_ = 200 V.

**Table 4:** Experimental parameter value.

Components	Part number	V/I rating
IGBTs	SKM 75GB063D	600 V/75 A
Diode	FX2000D	200 V/20 A
Controller	TI 28379D	200 MHz/32 bit
Driver circuit	TLP 250	I_F_ = 5 mA (max)
Floating capacitor	PG-6DI/200 V	2200 μF/200 V
Sensor	Current transformer	25 A
Resistive and inductive load	–	80 Ω and 100 mH/100 Ω and 80 mH
RC delay circuit	–	4 μs
Loop inductor	Core type	50 μH

In this prototype mode, the switching frequency and fundamental frequency is 2.5 kHz/50 Hz is used. Sudden load changes test the dynamic performance of the proposed topology. During this load changing, the corresponding waveforms are captured in DSO and presented in Fig. 6b for the load value from 80 Ω and 100 mH to 100 Ω and 80 mH with a load current of 5.4 A (pk–pk). Further, the disconnected load to 100 Ω and 80 mH is applied, and the corresponding waveforms are presented in Fig. 6c. Due to load variations, the modulation index will be adjusted in the closed-loop. The modulation index (*Ma*) is varied from 0.33 to 0.66/0.66 to 0.8 and 0.8–1.0, respectively and the respective output results are Fig. 6d–f, respectively. This *Ma* variation is done under the load value of 80 Ω and 100 mH. In all these conditions, the FCs voltage is not affected, and it maintains the voltage of 200 V. It confirms that the FC is independent of the load value. Further, the dynamic input variation from 100 to 200 V is applied, and the corresponding waveform is shown in Fig. 6g.

**Figure 7:**
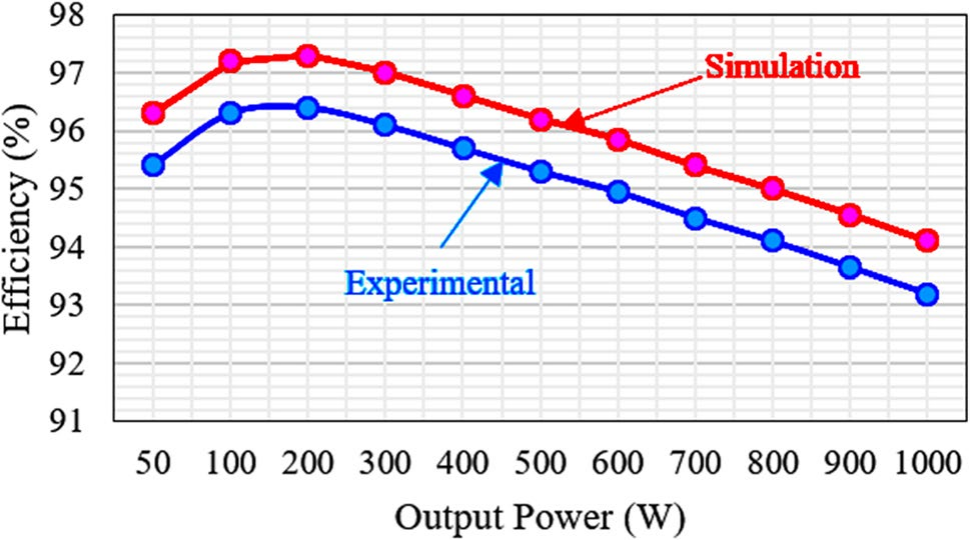
Output power vs efficiency of proposed RSC-SB2 topology.

**Figure 8:**
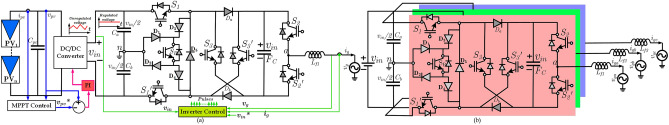
Proposed RSC-SB2 topology with (**a**) PV and simple grid control structure and (**b**) three-phase system extension.

Initially, the dc-link capacitors are charged to 100 V for *v*_*in*_ = 200 V, and FC is charged to 200 V. However, most of the Self-balanced FCs suffer from the inrush current. Therefore, a small loop inductor (50 µH) is used in the circuit to suppress the inrush current, and the switches *S*_1_ and *S*_1_′ are in the loop path with high inrush current. The switch voltage and current waveform of with and without loop inductor are shown in Fig. 6h,i. Further, to conclude the experimental section, the cost comparison of recent boost ANPC-type topologies and proposed is shown in Table 5, and the chosen IGBT device is half-bridge type. The proposed topology gives a lower cost as compared to the other topologies. The power loss for the individual component is calculated using PLECS simulation tool and the same is listed in Table 6. The cost of the proposed topology is compared with similar ANPC topologies as given in Table 7. It is confirming that the proposed topology is required low cost. Based on the power loss calculation, the efficiency versus output power is plotted in Fig. 7, and for the experimental efficiency is calculated using the Fluke meter. The application of the proposed topology is the PV system, as shown in Fig. 8a, and the possible three-phase extension is shown in Fig. 8b. The PV panel’s source-side ‘*n*’ is connected in series to meet the required grid voltage. The dc/dc converter is used to regulate the unregulated PV voltages and fed to the dc-link capacitors.

**Table 5:** Cost comparison of recent 7L boost ANPC type SCMLI topologies.

^a^Components/part number	V/I ratings	Unit price ($)	^8^	^9–11^	Prop
**IGBT** SKM 75GB063D	600 V/75 A	15.85	7	7	4
**Gate driver** TLP 250 board	2500 V	9.17‬	9	8	7
**FC capacitor** PG- 6DI/200 V	2200 μF/200 V	37.93	1	1	1
**Ultra-fast diode** RURG5060	600 V/50 A	3.42	–	–	7
Total cost ($)			231.41	222.24	189.46

^a^Mouser.com and tme.com.

**Table 6:** Power loss of each component for 80 Ω + 100 mH.

Components	S_1_	S_2_	S_3_	S_4_	S_5_	S_6_
Power loss (W)	2.666	0.475	2.668	0.5291	0.530	0.644

Components	S_7_	D_1_/D_2_	D_3_/D_4_	D_x_/D/D′	C_1_/C_2_	FC
Power loss (W)	0.645	0.2795	0.289	2.3465	0.072	5.725

**Table 7:** Cost comparison for proposed topology with other similar topologies^11–14^.

Part number	Rating	Unit price ($)	Proposed		^11^		^12^		^13,14^	
			Comp	Total price	Comp	Total price	Comp	Total price	Comp	Total price
**MOSFET switches**										
IRFP240PBF	200 V, 20 A	3.08	7	21.56	10	30.8	10	30.8	9	27.72
IRFP350PBF	400 V, 20 A	4.11	–	–	–	–	–	–	–	–
**Diodes**										
VI20200G-E3/4W	200 V, 20 A	1.8	7	12.6	–	–	–	–	–	–
**Gate driver circuits**										
HCPL-3120	15–30 V/V_IORM_ = 630 V	3.86	7	27.02	8	30.88	10	38.6	8	30.9
**Capacitors**										
EKMR201VSN102MP50S	200 V, 1.0 mF	4.5	–	–	–	–	–	–	–	–
EKMR201VSN222MR50S	200 V, 2.2 mF	6.07	1	6.07	–	–	–	–	–	–
E36D201MLS472TCA5M	200 V, 4.4 mF	10	–	–	2	20	1	10	1	10
Total price in USD			–	67.25	–	81.68		79.4	–	68.62

## Conclusion

A new 7L-RSC-SB2 inverter topology with reduced switch count and self-balanced and boosting ability topology was proposed in this letter. The output voltage is 1.5 times higher than the *v*_*in*_. The proposed topology used one floating capacitor, and the voltage stress on the individual switch is *v*_*in*_, reducing the inverter's overall voltage stress. The comprehensive analysis in terms of components and cost comparison is presented, and it is evident that the proposed topology is better than the other 7L SCMLI topologies. Further, the experimental results are validated, and the proposed boost type ANPC topology is a better alternative topology for the conventional ANPC inverter, and it's suitable for rooftop PV applications.

## Nomenclature

PV: Photovoltaic
NPC: Neutral point clamped
ANPC: Active neutral point clamped
*v*_*in*_: Input voltage
TL: Transformerless
SCMLI: Switched-capacitor multilevel inverter
FCs: Floating capacitors
IGBT: Insulated bipolar gate transistor
*v*_*o*_: Output voltage
Ma: Modulation index
i_c_: Charging current
i_o_: Output current
PWM: Pulse width modulation
C_opt_: Optimum capacitor value
